# Sequencing and Analysis of the Mediterranean Amphioxus (*Branchiostoma lanceolatum*) Transcriptome

**DOI:** 10.1371/journal.pone.0036554

**Published:** 2012-05-09

**Authors:** Silvan Oulion, Stephanie Bertrand, Mohamed R. Belgacem, Yann Le Petillon, Hector Escriva

**Affiliations:** CNRS, UMR7232, Université Pierre et Marie Curie Paris 06, Observatoire Océanologique, Banyuls-sur-Mer, France; Ecole Normale Supérieure de Lyon, France

## Abstract

**Background:**

The basally divergent phylogenetic position of amphioxus (Cephalochordata), as well as its conserved morphology, development and genetics, make it the best proxy for the chordate ancestor. Particularly, studies using the amphioxus model help our understanding of vertebrate evolution and development. Thus, interest for the amphioxus model led to the characterization of both the transcriptome and complete genome sequence of the American species, *Branchiostoma floridae*. However, recent technical improvements allowing induction of spawning in the laboratory during the breeding season on a daily basis with the Mediterranean species *Branchiostoma lanceolatum* have encouraged European Evo-Devo researchers to adopt this species as a model even though no genomic or transcriptomic data have been available. To fill this need we used the pyrosequencing method to characterize the *B. lanceolatum* transcriptome and then compared our results with the published transcriptome of *B. floridae*.

**Results:**

Starting with total RNA from nine different developmental stages of *B. lanceolatum*, a normalized cDNA library was constructed and sequenced on Roche GS FLX (Titanium mode). Around 1.4 million of reads were produced and assembled into 70,530 contigs (average length of 490 bp). Overall 37% of the assembled sequences were annotated by BlastX and their Gene Ontology terms were determined. These results were then compared to genomic and transcriptomic data of *B. floridae* to assess similarities and specificities of each species.

**Conclusion:**

We obtained a high-quality amphioxus (*B. lanceolatum*) reference transcriptome using a high throughput sequencing approach. We found that 83% of the predicted genes in the *B. floridae* complete genome sequence are also found in the *B. lanceolatum* transcriptome, while only 41% were found in the *B. floridae* transcriptome obtained with traditional Sanger based sequencing. Therefore, given the high degree of sequence conservation between different amphioxus species, this set of ESTs may now be used as the reference transcriptome for the *Branchiostoma* genus.

## Introduction

The Mediterranean amphioxus, *Branchiostoma lanceolatum*, belongs to the subphylum Cephalochordata, one of the three extant chordate groups with the urochordates and the vertebrates. Although urochordates are the closest relatives of vertebrates [1], they are extremely derived animals when considered from an anatomic and genomic viewpoint. In contrast, cephalochordates, which diverged earlier within the chordate lineage, show many conserved characteristics with vertebrates, such as their genomic organization, genetics, morphoanatomy and developmental mechanisms [2], [3]. Amphioxus is thus considered ‘vertebrate-like’, but simpler and characterized by a dorsal hollow nerve cord, dorsal notochord, ventral digestive tract, and pharynx perforated with gill slits. This relative simplicity is also found in its genome because cephalochordates diverged from the other chordates before the two whole genome duplication events that occurred at the base of the vertebrate diversification [4], [5]. For these reasons, amphioxus belongs to a phylogenetic group that is extremely important for understanding how vertebrates evolved from an invertebrate-chordate ancestor (i.e. Evo-Devo studies).

Three amphioxus species are currently used for Evo-Devo studies: the Floridian-Caribbean *Branchiostoma floridae*, the East Asian *B. belcheri*, and the Mediterranean *B. lanceolatum*. Even if the divergence time between these species was estimated to be between 100 and 200 Myr [6], [7] they show a high degree of conservation both at the morphological and gene expression levels [8]. *B. floridae* is to date the best-characterized species and its complete genome sequence [9], [10] as well as data for many ESTs [10], [11], [12] are published. However, the Mediterranean amphioxus species is becoming an attractive model since, unlike for the other species, a reliable method now exists for inducing spawning in the laboratory on a daily basis during the breeding season (i.e. from May to July) [13], [14]. *B. lanceolatum* is becoming a model for developmental biology and evolution [3], and its complete genome sequence will be available in the next future (http://www.genoscope.cns.fr/spip/L-espece-d-amphioxus-un-modele.html). In this context, in order to expand our knowledge and to develop new tools for the scientific community working with *B. lanceolatum*, we have sequenced and analyzed its normalized transcriptome. The high quality of the *B. lanceolatum* reference transcriptome obtained will become essential for the annotation and study of amphioxus genomic resources in future studies.

## Results and Discussion

### 454-pyrosequencing and Assembly

**Figure 1:**
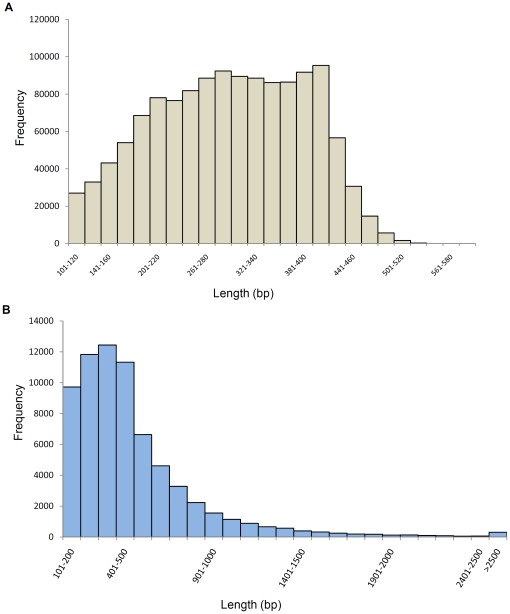
Size distribution of reads and contigs. Only reads (A) and contigs (B) bigger than 100 bp were figured.

**Figure 2:**
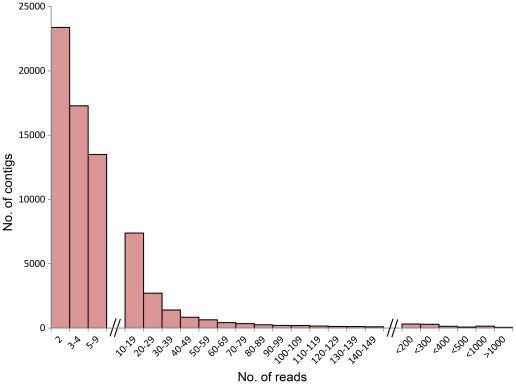
Composition of contigs. The majority of contigs have a low numbers of reads (less than 10 reads).

**Figure 3:**
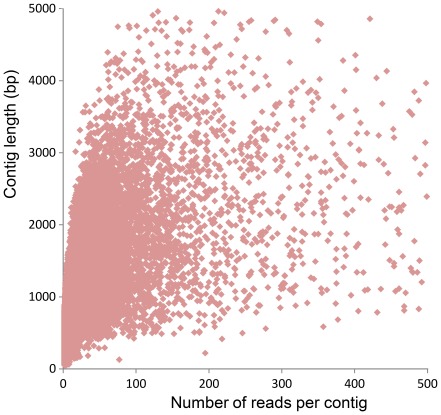
Scatter plot representing the number of reads per contig for each contig length.

A total of 1,423,403 reads (average length 275 bp) and 391 Mbp were generated by 454-pyrosequencing from a normalized random-primed cDNA library. The cDNA library was constructed with total RNA extracted from eight different developmental stages (eight-cell embryos, blastula, gastrula, four different neurula stages, and larva) as well as from adult tissues of the Mediterranean amphioxus, *B. lanceolatum* (Table 1; sequencing performed by GATC Biotech AG). After cleaning and removing the 5′ and 3′ 30 bp adapters of each sequence, a total of 1,148,112 high quality reads (318 Mbp) were obtained and used for *de novo* assembly. The size distribution of these high quality reads is shown in Figure 1A (sequences shorter than 100 bp were removed from the analysis). After clustering and assembly with the CLC workbench Version 4.6.1, a total of 70,530 contigs, whose size distribution is represented in Figure 1B, were obtained. Contig lengths ranged from 100 bp to 6,202 bp, with an average length of 490 bp (Table 1 and Figure 1B). The contig coverage ranges from 2 to more than 1,000 reads per contig, with the majority of contigs covered by less than 20 reads (Figure 2). There is a positive relationship between the length of a contig and the number of reads it contains (Figures 2 and 3), as expected for a randomly fragmented transcriptome.

**Table 1:** Sequence and assembly statistics.

Sequence statistics	
Raw sequencing reads	
*Number of reads*	1,423,403
*Total size, bp*	391,432,116
*Average size, bp*	275
Aligned reads	
*Number of reads*	1,153,224
*Total size, bp*	319,427,969
*Average size, bp*	277
*Maximum length, bp*	1175
*% GC*	44.2
*% N*	0.02
**Assembly statistics**	
*Number of contigs*	70,530
*Total size, bp*	34,583,174
*Average size of contigs, bp*	490
*Maximum length, bp*	6202
*% GC*	44.4

### Sequence Annotation

**Figure 4:**
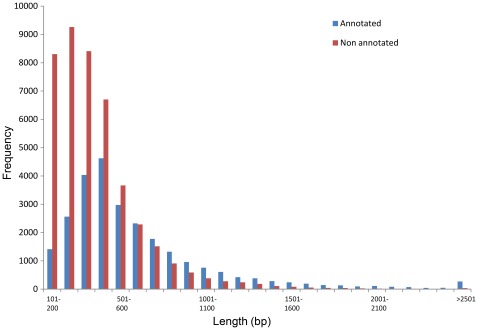
Size distribution of annotated (blue) and non-annotated (red) contigs. Non-annotated contigs are highly present within the shorter contigs (less than 500 bp).

The annotation of the 70,530 contigs was performed by BlastX searches against the SwissProt database and the NCBI non-redundant (nr) protein database using the Blast2GO suite [15], [16]. From the SwissProt database we have found matches for 17,930 sequences, and from the NCBI non-redundant (nr) protein database we obtained 7,964 supplementary matches. Thus, a total of 25,894 sequences were annotated (i.e. 37% of the contigs), and corresponded to 17,104 unique accession numbers. It is worth noting that the number of annotated short sequences (i.e. shorter than 500 bp) is less important than the number of annotated long sequences (i.e. longer than 500 bp) (Figure 4). This result can be explained by the presence in the short sequences of fragments corresponding to 5′ and 3′ UTRs that are not highly conserved between species and therefore not annotated.

If we consider that the Mediterranean amphioxus has the same gene set as the American species (i.e. about 21,900 protein coding loci [10]), we can estimate that the annotated subset of the *B. lanceolatum* transcriptome covers 78% (17,104 of 21,900) of the amphioxus genes. The majority of the top BLAST hits of these annotated sequences correspond to sequences from *Branchiostoma floridae.* This was expected since these two cephalochordate species are closely related and both the whole genome sequence and ESTs data are available for *B. floridae* in the databases used [9], [10], [11].

### Survey of the Transcriptome Representativeness

In order to validate the high presence of the amphioxus genes in the *B. lanceolatum* transcriptome (78%), we performed a survey of some multigenic families that have been well characterized in amphioxus previously. Thus, of the 132 homeobox genes identified in the *B. floridae* genome [17], 94 are present in the *B. lanceolatum* transcriptome (i.e. 71%) (Table S1). This result is similar to the 78% of coverage at the genome scale. Moreover, some genes (like the posterior Hox ones: from Hox7 to Hox15) that are not expressed in the eight developmental stages selected for the RNA pool used for the cDNA library construction, are not detected. Other well characterized superfamilies which are also well-represented in the *B. lanceolatum* transcriptome are the Fox gene family and the Nuclear Receptor (NR) gene family. We have identified 43 Fox genes of the 49 (i.e. 88%) described in *B. floridae* [18], and 25 nuclear receptors of the 33 (i.e. 76%) present in *B. floridae* [19], [20] (Table S2). These results confirm the good coverage of the amphioxus genome by the *B. lanceolatum* transcriptome. However, other gene families like the Fgf (Fibroblast growth factors) or the opsins are less present in the transcriptome. Indeed, only 2 of the 8 Fgfs [21] and 3 of the 20 opsins [9] are part of the *B. lanceolatum* transcriptome (Table S2). This low representation of some genes in the transcriptome may be explained by the low level of gene expression of some genes or because these genes are only expressed during late embryonic development (i.e. in late larva stages whose RNA was not included in the pool used for the cDNA library construction).

### Gene Ontology Analyses and Comparisons with the *B. floridae* Genomic and Transcriptomic Data

**Figure 5:**
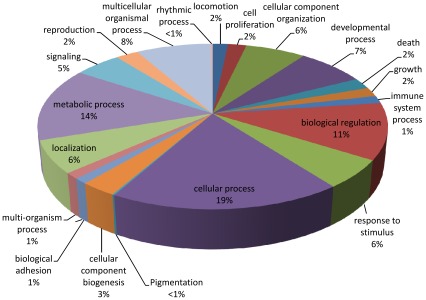
Biological Process. Gene Ontology (GO) assignment of the *Branchiostoma lanceolatum* transcriptome for the Biological Process category (total of 54,218 terms).

**Figure 6:**
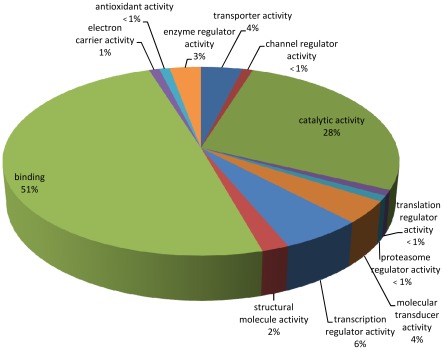
Molecular Function. Gene Ontology (GO) assignment of the *B. lanceolatum* transcriptome for the Molecular Function category (total of 85,213 terms).

**Figure 7:**
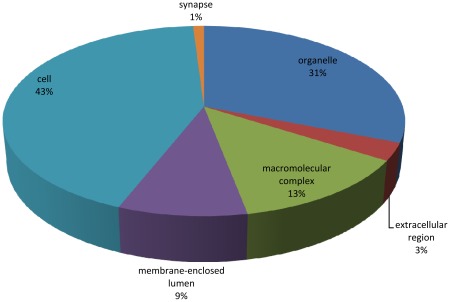
Cellular Component. Gene Ontology (GO) assignment of the *B. lanceolatum* transcriptome for the Cellular Component category (total of 48,754 terms).

Gene Ontology (GO) analyses of the *B. lanceolatum* transcriptome were performed using the Blast2GO suite annotation [22], [23], which provides information on the molecular function, the cellular component and the biological process for each sequence present in the transcriptome. In total, 188,185 GO terms were assigned for 21,819 (84%) sequences, classed into three independent ontology categories: (i) 54,218 (28.8%) terms corresponding to a biological process, (ii) 85,213 (45.3%) to a molecular function, and (iii) 48,754 (25.9%) to a cellular component. Level 2 of these GO assignments are shown in Figures 5, 6, and 7. Concerning biological processes, the most important categories present in the transcriptome are: cellular process (19%), metabolic process (14%) and biological regulation (11%), followed by multicellular organismal process (8%), developmental process (7%), cellular component organization (6%), response to stimulus (6%), localization (6%) and signaling (5%). Genes coding for other biological categories such as locomotion, growth, death, pigmentation and rhythmic processes are also present but in a lower proportion (Figure 5). Concerning the molecular function category, the binding (51%) and catalytic activities (28%) account for most of the terms, followed by transcription regulator (6%), molecular transducer (4%), transporter (4%) and enzyme regulator (3%) activities (Figure 6). Finally, among the cellular component category, 43% of the terms are related to the cell, 31% to organelles, 13% to macromolecular complexes, 9% to the membrane enclosed lumen, 3% to the extracellular region and 1% to the synapses (Figure 7).

The distribution of genes based on the GO terms within these three ontology categories in *B. lanceolatum* is consistent with a similar study carried out on *B. floridae* [10]. Indeed, in both species, the most represented terms corresponding to a biological process are cellular and metabolic processes as well as biological regulation. In addition, the binding and the catalytic activities are the major molecular functions; and the cell, organelle and macromolecular complexes are the main cellular components represented. Although there are a few differences within each category, the general organization and the main terms are similar in both amphioxus species. With the rapid rise of high throughput sequencing, many transcriptomic data are now available, in particular for vertebrate species. Comparing the transcriptome of both amphioxus species with other aquatic vertebrates (teleost fishes like the European eel [24], the rainbow trout [25], the guppy or the zebrafish [26]), we observed the same general organization for each ontological category of genes. Even if a possible bias may exist due to the huge amount of vertebrate transcriptomic data in databases, these results suggest that the observed gene content distribution in the presented amphioxus transcriptome is a common feature in chordates.

**Figure 8:**
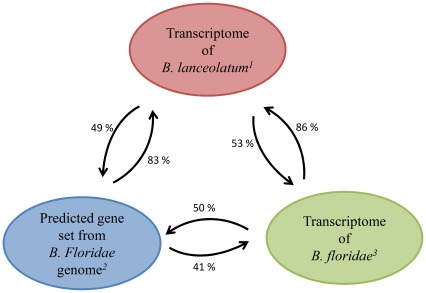
Genomic and transcriptomic comparisons between *B. floridae* and *B. lanceolatum*. BlastN were performed between each database in both directions. The arrow indicates the sense of the blast, from the query to the database. Databases used: (1) transcriptomic data of *B. lanceolatum* presented here; (2) genomic data of *B. floridae* published by Putnam et al. [10]; (3) transcriptomic data of *B. floridae* published by Yu et al. [11].

A comparative analysis (using BlastN approaches with an E-value cut-off of 1E^−3^ and a minimal alignment size of 50 bp) between the 70,530 contigs of *B. lanceolatum* and the genomic [10] and transcriptomic [11] data of *B. floridae* shows that 83% of the *B. floridae* predicted genes have significant hits for one or more sequences of the *B. lanceolatum* transcriptome (Figure 8). This is consistent with our previous data showing that the annotated part of the *B. lanceolatum* transcriptome covered 78% of the *B. floridae* predicted genes.

The 83% coverage of the amphioxus gene set obtained here is better than the coverage reported previously (only 41%) with the 262,037 ESTs of *B. floridae* [11] (Figure 8). The quality of both the assembly and the annotation of the *B. lanceolatum* transcriptome obtained in our present study confirm that high throughput sequencing on the GS FLX of a normalized cDNA library is better suited than traditional sequencing methods (i.e. Sanger) for acquiring an overview of a species’ transcriptomic landscape.

Additional differences between the two transcriptomic approaches are also evident when the two data sets are compared. For example, 86% of the *B. floridae* ESTs sequences (i.e. 226,250 of 262,037) have significant hits in the *B. lanceolatum* transcriptome, and only 53% of the *B. lanceolatum* transcriptome sequences (i.e. 37,034 of 70,530) match the *B. floridae* transcriptome (Figure 8). This result is supplementary evidence showing that the *B. lanceolatum* transcriptome set is larger than the *B. floridae* one, and confirms the robustness of this analysis. However, it is also fair to note that even if the coverage using a Sanger sequencing approach is lower, it results in a cDNA library whose clones are very useful for further studies.

However, comparing the *B. lanceolatum* transcriptome (annotated and non-annotated contigs) and the 21,900 predicted genes of the *B. floridae* genome found only 49% positive hits (34,560 of the 70,530 sequences). This result seems quite low even if it is consistent with 50% of the matches described for the same comparison using the *B. floridae* transcriptome [11] (Figure 8). Two possible explanations for this low number of hits can be suggested: first, the presence in the transcriptome of transcripts that do not code for proteins, and, second, the presence of short sequences corresponding to the non-translated regions of protein coding RNAs (5′ and 3′ UTRs). Both explanations are reasonable because the comparison between the annotated part of the *B. lanceolatum* transcriptome (i.e. 25,894 of the 70,530 assembled sequences) with the *B. floridae* predicted genes showed matches with 92% of the sequences and the majority of the annotated sequences of the transcriptome correspond to long sequences, while the shorter ones contain predominantly non-annotated sequences (Figure 4).

### Conclusions

In this study, RNA-seq was used to describe the reference transcriptome of the Mediterranean amphioxus *B. lanceolatum*. The strategy used here, combining the construction of a normalized cDNA library with RNAs extracted from nine key developmental stages with a high throughput GS FLX sequencing, resulted in the achievement of high quality results. Indeed, sequencing of 1,423,403 reads allowed their assembly into 70,530 contigs and the functional annotation of 37% of these. Moreover, the *B. lanceolatum* reference transcriptome contains more than 83% of the genes that have been predicted in the genome of the Floridian species. Given the morphological and functional closeness between different amphioxus species [8], as well as the sequence conservation of coding genes, these transcriptomic data should be extremely useful, not only for future research on *B. lanceolatum* but also on the other cephalochordate species. Moreover, these data will be essential as a basis for the characterization of the *B. lanceolatum* genome in the near future.

## Materials and Methods

### Amphioxus Sample, RNA Isolation and cDNA Library Construction

Ripe animals of the Mediterranean amphioxus species (*B. lanceolatum*) were collected from Argelès-sur-Mer (France), and gametes were obtained by heat stimulation [13], [14] (note that no specific permits were required for this study). *B. lanceolatum* embryos at different developmental stages (eight-cell embryos, blastula, gastrula, early neurula, mid-neurula, late neurula, neurula before the mouth opening and larva stages), as well as ripe adults, were frozen in liquid nitrogen. Total RNA was extracted using the RNeasy Plus Mini Kit (QIAGEN) after disrupting and homogenizing the sample with TissueLyser (QIAGEN). A mix of 25 µg of total RNA was used for the cDNA library construction by GATC Biotech SARL.

### Sequencing and Assembly

A cDNA library was constructed from isolated poly(A)+ RNA and normalized through denaturation and reassociation of cDNA according to standard protocols. Before sequencing, the library was PCR amplified (8 cycles) and gel purified (size range of interest: 500–800 bp). Sequencing was performed by GATC Biotech SARL using a GS FLX sequencer. The raw reads obtained were cleaned (by removing the adapters) and assembled by *de novo* assembling with the CLC workbench Version 4.6.1. Sequences were deposited in the NCBI Transcriptome Shotgun Assembly (TSA) database (accession numbers: JT846176 - JT905674).

### Functional Annotation

Functional annotation of the *B. lanceolatum* transcriptome was done using the Blast2GO software v.2.5.0 [15], [16]. Homology searches were first performed using BlastX against the SwissProt database. In a second step, queries that did not match with any SwissProt sequences were searched for using BlastX against the NCBI non-redundant protein database. Both BLAST searches were performed with the same parameters (E-value cut-off of 1E^−3^). For the Gene Ontology (GO) association to BLAST hits previously obtained, we used the Blast2GO suite with the following standard parameters: E-value <1E^−6^, annotation cut-off >55, and a GO weight >5.

## Supporting Information

Table S1 **Representativeness of Homeobox genes in the transcriptome.** Survey showing which amphioxus Homeobox genes are present in the *B. lanceolatum* transcriptome. The study was done on the homeobox genes identified by Takatori et al. [17], plus the related *Pon* and *Pax-1/9* genes. Green: the gene is present; red: the gene is absent.
(PDF)

Table S2 **Representativeness of Fox genes, Nuclear Receptor genes and Fibroblast Growth Factor genes in the transcriptome.** Survey showing which amphioxus Fox genes (A), Nuclear Receptor (NR) genes (B) and Fibroblast Growth Factor (Fgf) genes (C) are present in the *B. lanceolatum* transcriptome. Green: the gene is present; red: the gene is absent.
(PDF)

## References

[1] Delsuc F, Brinkmann H, Chourrout D, Philippe H (2006). Tunicates and not cephalochordates are the closest living relatives of vertebrates.. Nature.

[2] Schubert M, Escriva H, Xavier-Neto J, Laudet V (2006). Amphioxus and tunicates as evolutionary model systems.. Trends Ecol Evol.

[3] Bertrand S, Escriva H (2011). Evolutionary crossroads in developmental biology: amphioxus.. Development.

[4] Dehal P, Boore JL (2005). Two rounds of whole genome duplication in the ancestral vertebrate.. PLoS Biol.

[5] Kuraku S, Meyer A, Kuratani S (2009). Timing of genome duplications relative to the origin of the vertebrates: did cyclostomes diverge before or after?. Mol Biol Evol.

[6] Canestro C, Albalat R, Hjelmqvist L, Godoy L, Jornvall H (2002). Ascidian and amphioxus Adh genes correlate functional and molecular features of the ADH family expansion during vertebrate evolution.. J Mol Evol.

[7] Nohara M, Nishida M, Manthacitra V, Nishikawa T (2004). Ancient phylogenetic separation between Pacific and Atlantic cephalochordates as revealed by mitochondrial genome analysis.. Zoolog Sci.

[8] Somorjai I, Bertrand S, Camasses A, Haguenauer A, Escriva H (2008). Evidence for stasis and not genetic piracy in developmental expression patterns of Branchiostoma lanceolatum and Branchiostoma floridae, two amphioxus species that have evolved independently over the course of 200 Myr.. Dev Genes Evol.

[9] Holland LZ, Albalat R, Azumi K, Benito-Gutierrez E, Blow MJ (2008). The amphioxus genome illuminates vertebrate origins and cephalochordate biology.. Genome Res.

[10] Putnam NH, Butts T, Ferrier DE, Furlong RF, Hellsten U (2008). The amphioxus genome and the evolution of the chordate karyotype.. Nature.

[11] Yu JK, Wang MC, Shin IT, Kohara Y, Holland LZ (2008). A cDNA resource for the cephalochordate amphioxus Branchiostoma floridae.. Dev Genes Evol.

[12] Jin P, Ji X, Wang H, Li-Ling J, Ma F (2010). AmphiEST: Enabling comparative analysis of ESTs from five developmental stages of amphioxus.. Mar Genomics.

[13] Fuentes M, Benito E, Bertrand S, Paris M, Mignardot A (2007). Insights into spawning behavior and development of the European amphioxus (Branchiostoma lanceolatum).. J Exp Zool B Mol Dev Evol.

[14] Fuentes M, Schubert M, Dalfo D, Candiani S, Benito E (2004). Preliminary observations on the spawning conditions of the European amphioxus (Branchiostoma lanceolatum) in captivity.. J Exp Zool B Mol Dev Evol.

[15] Gotz S, Garcia-Gomez JM, Terol J, Williams TD, Nagaraj SH (2008). High-throughput functional annotation and data mining with the Blast2GO suite.. Nucleic Acids Res.

[16] Conesa A, Gotz S, Garcia-Gomez JM, Terol J, Talon M (2005). Blast2GO: a universal tool for annotation, visualization and analysis in functional genomics research.. Bioinformatics.

[17] Takatori N, Butts T, Candiani S, Pestarino M, Ferrier DE (2008). Comprehensive survey and classification of homeobox genes in the genome of amphioxus, Branchiostoma floridae.. Dev Genes Evol.

[18] Yu JK, Mazet F, Chen YT, Huang SW, Jung KC (2008). The Fox genes of Branchiostoma floridae.. Dev Genes Evol.

[19] Schubert M, Brunet F, Paris M, Bertrand S, Benoit G (2008). Nuclear hormone receptor signaling in amphioxus.. Dev Genes Evol.

[20] Langlois MC, Vanacker JM, Holland ND, Escriva H, Queva C (2000). Amphicoup-TF, a nuclear orphan receptor of the lancelet Branchiostoma floridae, is implicated in retinoic acid signalling pathways.. Dev Genes Evol.

[21] Bertrand S, Camasses A, Somorjai I, Belgacem MR, Chabrol O (2011). Amphioxus FGF signaling predicts the acquisition of vertebrate morphological traits.. Proc Natl Acad Sci U S A.

[22] The Gene Ontology Consortium (2004). The Gene Ontology Consortium: The Gene Ontology (GO) database and informatics resource.. Nucleic Acids Res.

[23] The Gene Ontology Consortium (2008). The Gene Ontology Consortium: The Gene Ontology project in 2008.. Nucleic Acids Res.

[24] Coppe A, Pujolar JM, Maes GE, Larsen PF, Hansen MM (2010). Sequencing, de novo annotation and analysis of the first Anguilla anguilla transcriptome: EeelBase opens new perspectives for the study of the critically endangered European eel.. BMC Genomics.

[25] Salem M, Rexroad CE, Wang J, Thorgaard GH, Yao J (2010). Characterization of the rainbow trout transcriptome using Sanger and 454-pyrosequencing approaches.. BMC Genomics.

[26] Fraser BA, Weadick CJ, Janowitz I, Rodd FH, Hughes KA (2011). Sequencing and characterization of the guppy (Poecilia reticulata) transcriptome.. BMC Genomics.

