# Supplementary material for: Sequencing and Analysis of the Mediterranean Amphioxus (Branchiostoma lanceolatum) Transcriptome
Source: PLoS One. 2012 May 9;7(5):e36554. doi: 10.1371/journal.pone.0036554 (PMC3348903; doi:10.1371/journal.pone.0036554)
Supplement: Table S1 — Representativeness of Homeobox genes in the transcriptome. Survey showing which amphioxus Homeobox genes are present in the B. lanceolatum transcriptome. The study was done on the homeobox genes identified by Takatori et al. [17], plus the related Pon and Pax-1/9 genes. Green: the gene is present; red: the gene is absent. (PDF) [file pone.0036554.s001.pdf]

Class	Gene family	Gene name	
ANTP	En	En	x
	Gbx	Gbx	x
	Mnx	Mnxa	x
		Mnxb	x
	Evx	Evxa	x
		Evxb	x
	Hox	Hox1	x
		Hox2	o
		Hox3	x
		Hox4	x
		Hox5	o
		Hox6	x
		Hox7	o
		Hox8	o
		Hox9	o
		Hox10	o
		Hox11	o
		Hox12	o
		Hox13	o
		Hox14	o
		Hox15	o
	ParaHox	Gsx	o
		Xlox	o
		Cdx	x
	Meox	Mox	x
	Ro	Ro	o
	Barh	Barh	x
	Barx	Barx	x
	Bari	Bari	o
	Bsx	Bsx	o
	Dbx	Dbx	x
	Dlx	Dll	x
	Emx	Emxa	x
		Emxb	x
	Hlx	Hlx	o
	Hx	Hx	x
	Msx	Msx	x
	Msxlx	Msxlx	o
	Nk2.1/Scro	Nkx2-1	x
	Nk2.2/Vnd	Nkx2-2	x
	Hmx	Hmx	x
	Nk6	Nkx6	x
	Nk7	Nkx7	x
	Noto	Not	x
	Vax	Vax	o
	Ventx	Vent1	x
		Vent2	x
	Lcx	Lcx	x
	Lb	Lbx	x
	Nk1/Slou	Nkx1a	o
		Nkx1b	o
	Nk3/Bap	Nkx3	x
	Nk4/Tin/Csx	Csx	x
	Tlx	Tlx	x
	Nedx	Nedxa	x
		Nedxb	x
	Abox	Abox	o
	Ankx	Ankx	x
	Hhex	Hhex	x

Class	Gene family	Gene name	
PRD	Pax1/9	Pax1/9	x
	Pax2/5/8	Pax2/5/8	x
	Pax3/7	Pax3/7	x
	Pax4/6	Pax6	x
	Pon	Pon	o
	Arx	Arx	o
	Alx	Alx	x
	Vsx	Vsx	x
	Isx	Isx	x
	Dmbx	Dmbx	x
	Drgx	Drgx	o
	Gsc	Gsc	x
	APrdA	Aprd1	o
	APrdB	Aprd2	x
	APrdC	Aprd3	o
	APrdD	Aprd4	x
		Aprd5	x
	APrdE	Aprd6	x
	Otp	Otp	x
	Otx	Otx	x
	Phox	Phox	o
	Prop	Prop	o
	Pitx	Pitx	x
	Prrx	Prrx	o
	Repo	Repo	o
	Rax	Rax	o
	Shox	Shox	x
	Uncx	UncxA	x
		UncxB	x
		UncxC	o
	Hopx	Hopx	x
CUT	Onecut	Onecut	x
	Cux	Cux	x
	Cmp	Compass	x
	Acut	Acut	x
LIM	Lhx2/9	Lhx2/9-a	x
		Lhx2/9-b	o
	Isl	Isl	x
	Lhx1/5	Lhx1/5	x
	Lhx3/4	Lhx3/4	x
	Lhx6/8	Lhx6/8	o
	Lmx	Lmx	x
PROS	Prox	Prox	x
SINE	Six1/2	Six1/2	x
	Six3/6	Six3/6	x
	Six4/5	Six4/5	x
TALE	Irx	IrxA	x
		IrxB	x
		IrxC	x
	Meis	Meis	x
	Mkx	Mkx	x
	Atale	Atale	o
	Pbx	Pbx	x
	Pknox	Pknox	x
	Tgif	Tgif	x

Class	Gene family	Gene name	
ZF	Zfhx	Zfhx	x
	Azfh	Azfh	x
	Zeb	Zeb	x
	Tshz	Tshz	x
	Zhx	Zhx	x
CERS	Cers	Cers	x
POU	POU1	POU1	o
	POU2	POU2	x
	POU3	POU3	x
	POU4	POU4	x
	POU6	POU6	x
	POU3	POU3L	x
	Hdx	Hdx	x
HNF	HNF1	Tcf	x
	Hmbox	Hmbox1A	x
		Hmbox1B	x
	Ahnfx	Ahnf	x
Other	Ahbx	Ahbx1	x
	Muxa	Muxa	o
	Muxa	Muxb	x

Additional table 1
