# Supplementary material for: Sequencing and Analysis of the Mediterranean Amphioxus (Branchiostoma lanceolatum) Transcriptome
Source: PLoS One. 2012 May 9;7(5):e36554. doi: 10.1371/journal.pone.0036554 (PMC3348903; doi:10.1371/journal.pone.0036554)
Supplement: Table S2 — Representativeness of Fox genes, Nuclear Receptor genes and Fibroblast Growth Factor genes in the transcriptome. Survey showing which amphioxus Fox genes (A), Nuclear Receptor (NR) genes (B) and Fibroblast Growth Factor (Fgf) genes (C) are present in the B. lanceolatum transcriptome. Green: the gene is present; red: the gene is absent. (PDF) [file pone.0036554.s002.pdf]

A

Class	Gene name	
**FoxA**	FoxAa	x
	FoxAb	x
**FoxB**	FoxB	x
**FoxAB**	FoxABA	o
	FoxABB	o
**FoxC**	FoxC	x
**FoxD**	FoxD	x
**FoxE**	FoxEaA	x
	FoxEaB	x
	FoxEb	x
	FoxEd	x
	FoxEe	x
	FoxEc	x
	FoxEf	x
	FoxEg	x
	FoxEh	x
	FoxEi	x
**FoxF**	FoxF	x
**FoxG**	FoxG	x
**FoxH**	FoxHA	x
	FoxHB	x
**FoxI**	FoxI	x
**FoxJ1**	FoxJ1A	x
	FoxJ1B	x
**FoxJ2/3**	FoxJ2/3	x
**FoxK**	FoxKA	x
	FoxKB	x
**FoxL1**	FoxL1	x
**FoxL2**	FoxL2A	o
	FoxL2B	x
**FoxM**	FoxMA	x
**FoxN1/4**	FoxN1/4bA	x
	FoxN1/4bB	x
	FoxN1/4aA	x
	FoxN1/4aB	x
**Fox2/3**	FoxN2/3A	x
	FoxN2/3B	x
**FoxO**	FoxOA	o
	FoxOB	o
**FoxP**	FoxPA	x
	FoxPB	x
**FoxQ1**	FoxQ1	x
**FoxQ2**	FoxQ2aA	x
	FoxQ2aB	x
	FoxQ2bA	x
	FoxQ2bB	x
	FoxQ2c	x
**Fox1**	Fox1A	o
	Fox1B	x

B

	NR group	Gene name	
**NR0**	NR0B	NR0B	x
**NR1**	NR1A	TR	x
	NR1B	RAR	x
	NR1C	PPAR	o
	NR1D	REV-ERB	x
	NR1F	ROR	x
	NR1H	NR1H-1	x
	NR1H	NR1H-2	x
	NR1H	NR1H-3	x
	NR1H	NR1H-4	o
	NR1H	NR1H-5	x
	NR1H	NR1H-6	x
	NR1H	NR1H-7	x
	NR1H	NR1H-8	x
	NR1H	NR1H-9	x
	NR1H	NR1H-10	x
**NR2**	NR2A	HNF4	x
	NR2B	RXR	x
	NR2C	TR2/4	x
	NR2D	COUP-TF	x
	NR2E	TLX	o
	NR2E	PNR	o
	NR2E	NR2E	x
**NR3**	NR3A	ER	x
	NR3B	ERR	x
	NR3C	SR	o
**NR4**	NR4A	NR4A	x
**NR5**	NR5A	NR5A	o
	NR5B	NR5B	o
**NR6**	NR6A	GCNF	x
	NRa	NRa	x
	NRb	NRb	o
	NRc	NRc	x

C

	Gene name	
**Fgf**	Fgf1/2	o
	Fgf8/17/18	x
	Fgf9/16/20	o
	FgfA	o
	FgfB	o
	FgfC	x
	FgfD	o
	FgfE	o

Additional table 2
